# Neonatal Listeriosis with Central Nervous System Involvement: A Case Series and Review of the Literature

**DOI:** 10.3390/antibiotics15020206

**Published:** 2026-02-13

**Authors:** Chiara Maddaloni, Ludovica Martini, Domenico Umberto De Rose, Daniela Longo, Alessia Guarnera, Cinzia Auriti, Francesca Campi, Maria Paola Ronchetti, Andrea Dotta

**Affiliations:** 1 Neonatal Intensive Care Unit, Bambino Gesù Children’s Hospital, IRCCS, 00165 Rome, Italy; chiara.maddaloni@opbg.net (C.M.); ludovica.martini@opbg.net (L.M.); francesca.campi@opbg.net (F.C.); mariapaola.ronchetti@opbg.net (M.P.R.); andrea.dotta@opbg.net (A.D.); 2 Functional and Interventional Neuroradiology Unit, Bambino Gesù Children’s Hospital, IRCCS, 00165 Rome, Italy; daniela.longo@opbg.net (D.L.); alessia.guarnera@opbg.net (A.G.); 3 Medicine Interdipartimental Faculty, Saint Camillus International University of Health and Medical Sciences, 00131 Rome, Italy; cinzia.auriti@gmail.com; 4 Neuroradiology Unit, NESMOS Department Sant’Andrea Hospital, La Sapienza University, Via di Grottarossa, 1035–1039, 00189 Rome, Italy

**Keywords:** *Listeria monocytogenes*, sepsis, meningitis, infant, newborn, magnetic resonance imaging, intrathecal injections, case series, narrative review

## Abstract

*Background: Listeria monocytogenes* infection during pregnancy remains an underrecognized cause of severe neonatal disease, frequently leading to central nervous system (CNS) involvement with high mortality and long-term neurological sequelae. *Case presentation:* We report a case series of four neonates with confirmed neonatal listeriosis and neurological complications, managed in a tertiary neonatal intensive care unit (NICU). Clinical features, microbiological findings, neuroimaging, treatments, and outcomes were analyzed. Our cases presented with early-onset disease and severe clinical courses, including sepsis, meningitis, ventriculitis, hydrocephalus, and seizures. Neuroimaging revealed extensive CNS injury, ranging from intraventricular hemorrhage to multiloculated hydrocephalus. Outcomes varied from near-normal neurodevelopment to profound neurological impairment, despite appropriate antimicrobial therapy. A narrative review of previous cases of neonatal listeriosis was also performed to contextualize our findings. *Conclusions:* Neonatal listeriosis remains associated with severe neurological morbidity. Early recognition, advanced neuroimaging, multidisciplinary management, and preventive maternal strategies are essential to improve outcomes.

## 1. Introduction

*Listeria monocytogenes* infection during pregnancy remains a significant but often underrecognized threat, with the potential to cause severe early-onset neonatal disease despite advances in perinatal care [1,2].

Maternal listeriosis frequently presents as a mild, flu-like illness in pregnant women, yet it can lead to devastating consequences such as fetal demise, preterm labor, or neonatal sepsis and meningitis [3,4]. Indeed, fetal and neonatal infections may be severe, resulting in high rates of mortality and morbidity [5].

Improving maternal awareness and adherence to preventive measures is essential to reduce the risk of transplacental transmission [6]. *L. monocytogenes* is notorious for its affinity for the placenta and its ability to cross the maternal–fetal barrier [6]. Public health guidelines recommend that pregnant women avoid high-risk foods such as unpasteurized milk and cheeses, deli meats, and unwashed produce, and adopt strict hygiene practices in food preparation [7,8].

Neonatal disease can manifest as early-onset sepsis within the first 24–72 h of life or as late-onset meningitis in the second to fourth week of life [9]. Management of neonatal listeriosis, particularly when complicated by central nervous system involvement, requires a highly coordinated, multidisciplinary approach involving infectious disease specialists, neurologists, and neurosurgeons. The case fatality rate remains high, and survivors often endure serious neurological and developmental sequelae [10].

Here, we present four cases of neonatal listeriosis with neurological involvement. This case series was conducted and reported in accordance with the CARE (CAse REport) guidelines. The combination of severe infectious complications and extensive neurological injury can result in profoundly impaired neurodevelopmental outcomes, underscoring the critical importance of prevention, early recognition, and comprehensive specialized care. A brief review of the literature about a previously described case of neonatal listeriosis is also provided. The diagnostic workup, guided by laboratory results and neuroimaging, led to targeted antibiotic therapy and multidisciplinary management. Prevention through maternal education, prompt diagnosis, and early, aggressive treatment remains the cornerstone of improving outcomes in perinatal listeriosis.

## 2. Clinical Cases

**Figure 1:**
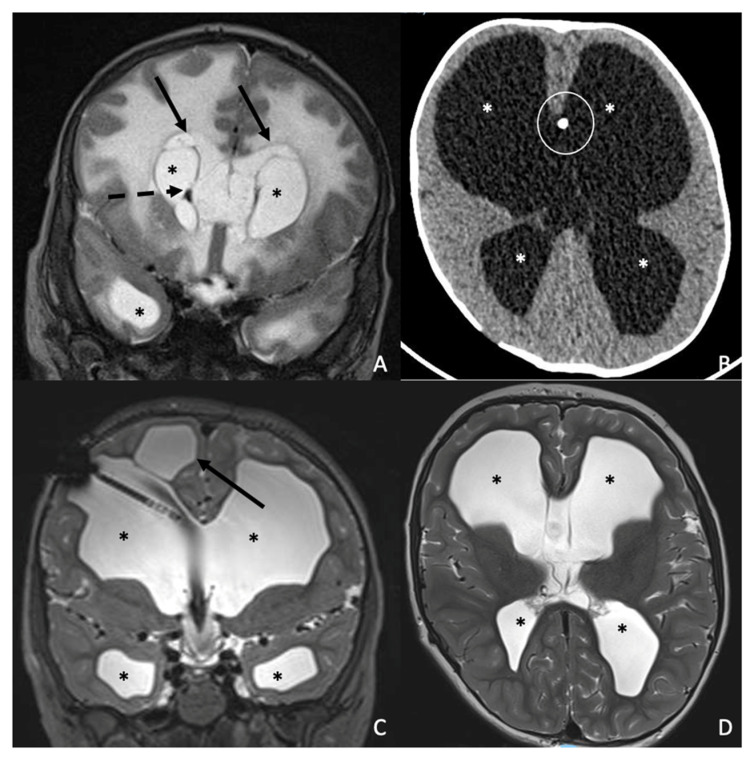
Serial neuroimaging studies in a boy diagnosed with *Listeria monocytogenes* infection. The figure includes the first MRI performed at 1 month and 10 days of age (**A**), followed by a CT scan at 1 month and 20 days of age (**B**), and the final MRI at 7 years of age (**C**,**D**). Baseline coronal T2-weighted imaging (**A**) shows diffuse supratentorial cortical and white-matter edema with effacement of the subarachnoid spaces at the convexities and skull base. There is marked dilatation of the lateral ventricles and third ventricle (black asterisks), with parietal ventricular-margin susceptibility consistent with hemorrhagic products/hemosiderin deposition (black dotted arrow), and multiple periventricular and deep white-matter cystic dilatations (black arrows). The non-contrast axial CT (**B**) shows further ventricular dilatation consistent with worsening hydrocephalus (white asterisks) and the presence of a ventriculoperitoneal shunt catheter (white circle). Follow-up MRI at 7 years of age includes coronal (**C**) and axial (**D**) T2-weighted images, showing persistent ventricular dilatation (black asterisks) with effacement of the subarachnoid spaces at the convexities and skull base, as well as the ventriculoperitoneal shunt catheter and multiple periventricular and deep white-matter cystic dilatations (black arrow).

**Case 1:** The patient was a full-term male newborn (39 + 2 weeks) delivered by cesarean section due to pathological fetal monitoring. Birth weight was 3140 g. Maternal anamnesis was unremarkable. On the 2nd day of life, he developed sepsis with respiratory failure, and on day 4, he presented a generalized seizure. Lumbar puncture confirmed *Listeria monocytogenes* in the cerebrospinal fluid. The brain MRI performed at 1 month and 10 days of age showed diffuse supratentorial cortical and white-matter edema with effacement of the subarachnoid spaces at the convexities and skull base, associated with marked dilatation of the lateral ventricles and third ventricle, parietal ventricular-margin susceptibility consistent with hemorrhagic products/hemosiderin deposition and multiple periventricular and deep white-matter cystic dilatations (Figure 1A). He was treated with ampicillin (100 mg/kg every 8 h) for 21 days and gentamicin (4 mg/kg every 24 h) for 14 days. He was transferred on day 35 of life to our neonatal intensive care unit (NICU) for continuation of care, where he was managed for post-infectious hydrocephalus and choanal stenosis. Due to progressive ventricular dilation, he underwent endoscopic third ventriculocisternostomy at 3 months of age, with satisfactory postoperative stability. Multiple procedures were performed for choanal stenosis, resulting in clinical improvement. One month after discharge, cranial ultrasound and head circumference measurements suggested progression of hydrocephalus, prompting readmission. At 5 months of age, following completion of the preoperative diagnostic evaluation and acquisition of a preoperative head computed tomography (CT) scan, the patient underwent placement of a right ventriculoperitoneal shunt (VP) with an adjustable valve set at 12 cm H_2_O. The postoperative course was uneventful. A sleep–wake electroencephalogram (EEG) performed during hospitalization did not show epileptiform abnormalities. At 6 months, the patient developed VP shunt malfunction, complicated by bowel perforation and migration of the distal catheter into the rectal ampulla, requiring appendectomy and placement of a new right VP shunt with the same valve setting. He continued annual magnetic resonance imaging (MRI) and neurosurgical follow-up, with imaging consistently showing stable ventricular size and good intracranial compensation. Particularly, the last follow-up MRI performed at 7 years of age showed persistent and stable ventricular dilatation with effacement of the subarachnoid spaces at the convexities and skull base, as well as the ventriculoperitoneal shunt catheter and multiple periventricular and deep white-matter cystic dilatations (Figure 1C,D). From a neurological standpoint, beginning at 9 years of age, he experienced sporadic transient episodes characterized by brief impaired responsiveness, pallor, and occasional focal motor manifestations. The frequency of these episodes progressively decreased after initiation of carbamazepine therapy, and no persistent neurological deficits developed. EEG abnormalities gradually stabilized. He attends school with learning support. At the most recent evaluation (12 years), he demonstrated stable neurosurgical status, normal laboratory results (carbamazepine level within range), adequate shunt function, and overall good neurological performance. He engages in regular physical activity, including basketball, and maintains a normal sleep–wake cycle.

**Figure 2:**
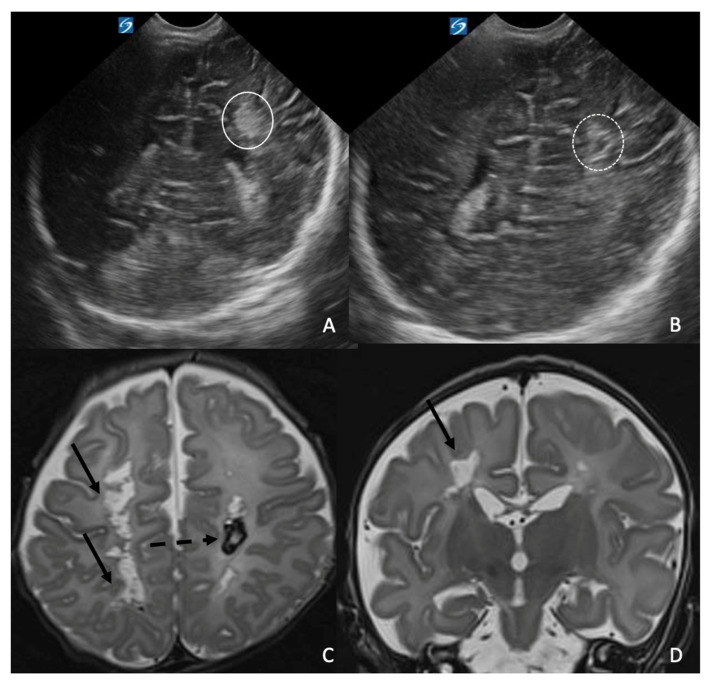
Brain USs performed at 10 and 20 days of life and the brain MRI performed at 1 month of age in a girl diagnosed with *Listeria monocytogenes* infection. The coronal US Images show the presence of a hyperechoic hemorrhagic focus (white circle in (**A**)), which showed a subsequent hypoechoic core, consistent with progressive resorption (dotted white circle in (**B**)). The axial (**C**) and coronal (**D**) T2WI sequences show multiple bilateral periventricular and centrum semiovale cystic lesions (black arrows) distributed along the course of the deep medullary veins, some of which demonstrate the presence of blood products (dotted black arrow in (**C**)).

**Case 2:** The second patient was a full-term female newborn (37 + 2 weeks) delivered by cesarean section for podalic presentation. Birth weight was 2830 g. Maternal anamnesis was unremarkable. Initial adaptation was normal, with Apgar scores of 9 and 10. At 12 h of life, she developed respiratory distress and hypo-reactivity, requiring escalation from low-flow oxygen to nasal continuous positive pressure (CPAP). Laboratory studies revealed increasing inflammatory markers, and empiric therapy with ampicillin–sulbactam and gentamicin was initiated. By the second day of life, her respiratory condition deteriorated, with echocardiographic evidence of right heart enlargement and elevated pulmonary artery pressure. Treatment with norepinephrine, dobutamine, and inhaled nitric oxide was started. Blood cultures grew *Listeria monocytogenes*, and antibiotic therapy was adjusted to high-dose ampicillin–sulbactam (100 mg/kg every 12 h) plus vancomycin (10 mg/kg every 12 h). Progressive respiratory and hemodynamic instability required endotracheal intubation on day 4. Cardiology evaluation confirmed severe pulmonary hypertension, a large patent ductus arteriosus, and a right-to-left shunt; milrinone was initiated. Due to refractory shock and respiratory failure, the infant was transferred to a tertiary center for extracorporeal membrane oxygenation (ECMO) support at 6 days of life. During ECMO, she received maximal-dose ampicillin (100 mg/kg every 8 h) and gentamicin (4 mg/kg every 24 h) as standard therapy for Listeria infection, with meropenem (20 mg/kg every 12 h) added due to increased ECMO-related infectious risk. Anticoagulation and severe thrombocytopenia contraindicated lumbar puncture. She was successfully decannulated from ECMO after 11 days and subsequently transferred back to our NICU. Respiratory status improved steadily, allowing extubation 7 days later, followed by non-invasive ventilation and ultimately spontaneous breathing at 27 days of life. Cardiac parameters gradually normalized, enabling tapering and discontinuation of sildenafil. Inflammatory markers steadily decreased, and antimicrobial therapy was completed after a 21-day course. Nutritional support progressed from parenteral to full enteral feeding by bottle, with good tolerance and appropriate weight gain. Audiologic and ophthalmologic evaluations were normal. Serial cranial ultrasounds were performed from birth. At 10 days of life, hyperechoic lesions with fuzzy margins were identified in the left deep parietal region and the right frontal horn. These lesions were consistent with hemorrhagic foci, which increased in size until the brain US performed at 20 days of age, which showed size stability with progressive resorption of the lesions (Figure 2A,B). The brain MRI performed at 1 month of age showed multiple bilateral periventricular and centrum semiovale cystic lesions distributed along the course of the deep medullary veins, some of which demonstrated the presence of blood products (Figure 2B,C). Electrophysiologic studies showed a disorganized EEG background, poor bilateral Brainstem Auditory Evoked Potentials (BAEP) wave V morphology, and absent bilateral cortical N20 responses on Somatosensory Evoked Potentials (SSEPs). Neurological examination revealed poor spontaneous movement, axial hypotonia with limb hypertonia, and delayed postural responses. Neurodevelopmental physiotherapy was initiated. The infant was unfortunately lost to follow-up after hospital discharge.

**Figure 3:**
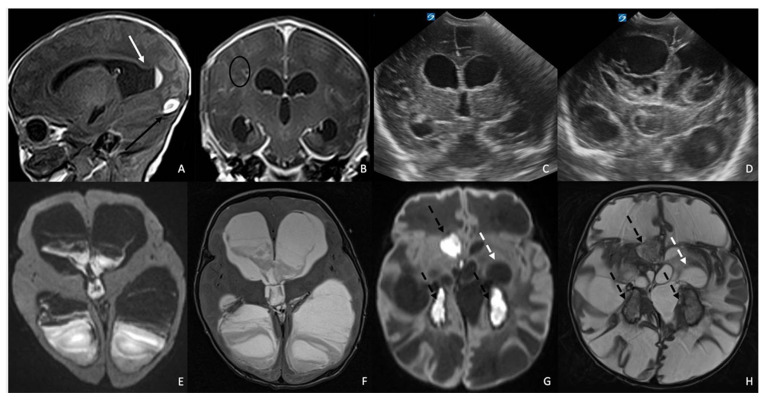
Serial brain MRIs performed at 10 days (**A**,**B**), 2 months and 1 week (**E**,**F**), and 4 months of age (**G**,**H**), and serial brain USs performed at 1 (**C**) and 2 (**D**) months of age in a boy diagnosed with *Listeria monocytogenes* infection. Baseline pre-contrast T1WI shows ventricular dilatation, intraventricular hemorrhage (white arrow in (**A**)), and thrombosis of the right transverse sinus (black arrow in (**A**)). Post-contrast T1WI confirms ventricular dilatation and demonstrates linear enhancement of the ventricular walls, consistent with ventriculitis, as well as meningeal enhancement consistent with meningitis. Focal hypointense lesions with peripheral enhancement are evident in the right parietal periventricular tissue, consistent with parenchymal abscesses (black oval in (**B**)). Coronal US images demonstrate initial ventricular dilatation (**C**), progressing to further enlargement of the ventricular system filled with inhomogeneously hyperechoic material (**D**). MRI at 2 months and 1 week includes axial DWI (**E**) and T2WI (**F**), showing increased ventricular dilation and purulent material within the ventricular system, appearing as inhomogeneous areas of diffusion restriction on DWI (**E**) and irregular foci of hypointensity within the T2-hyperintense ventricles (**F**). Follow-up MRI at 4 months includes axial DWI (**G**) and T2WI (**H**), demonstrating a marked decrease in ventricular size, with septated ventricles containing residual purulent material that shows intense diffusion restriction on DWI and irregular areas of ventricular hypointensity on T2WI (dotted black arrows in (**G**,**H**)), associated with diffuse white-matter hyperintensity and cystic degeneration (dotted white arrows in (**G**,**H**)).

**Case 3:** The third patient was a male neonate born at 37 weeks and 3 days of gestational age via emergency cesarean section due to abnormal cardiotocographic (CTG) findings. The mother was in her third pregnancy with no relevant antenatal complications; her maternal history was absent. At birth, Apgar scores were 5 at 1 min and 8 at 5 min. Birth weight was 3030 g. The newborn presented with bradycardia, hypotonia and hypo reactivity requiring positive pressure ventilation (FiO_2_ 30%) and subsequent non-invasive respiratory support with High-Flow Nasal Cannula (HFNC), for the first three days of life. At approximately 12 h of life, the newborn developed seizures characterized by upper-limb hypertonia, nystagmus, and oxygen desaturations, requiring treatment with an intravenous phenobarbital loading and maintenance dose, followed by intravenous midazolam. EEG confirmed epileptic activity. Blood culture and cerebrospinal fluid analysis were positive for *Listeria monocytogenes*, and intravenous ampicillin (100 mg/kg every 8 h) plus gentamicin (4 mg/kg every 24 h) was promptly initiated. The initial cranial ultrasound was unremarkable. Despite appropriate antimicrobial therapy, subsequent neuroimaging revealed progressive central nervous system involvement. Serial cranial ultrasounds demonstrated intraventricular hemorrhage and progressive ventricular dilatation. The MRI performed at 10 days of age showed ventricular dilatation, intraventricular hemorrhage, and thrombosis of the torcular herophilus and the right transverse sinus. Post-contrast T1WI confirms ventricular dilatation and demonstrates linear enhancement of the ventricular walls, consistent with ventriculitis, as well as meningeal enhancement consistent with meningitis. In the right parietal periventricular tissue, focal hypointense lesions with peripheral enhancement were consistent with parenchymal abscesses (Figure 3A,B). The infant was transferred to our NICU on day 11 of life. Progressive hydrocephalus required placement of a right endoventricular catheter with a Rickham reservoir for serial cerebrospinal fluid drainage. Although microbiological clearance of *Listeria monocytogenes* was achieved after 21 days of targeted therapy, the clinical course was complicated by secondary Gram-negative sepsis, meningitis and ventriculitis caused by ESBL-producing *Klebsiella pneumoniae*, requiring prolonged systemic antibiotic therapy (meropenem at a dosage of 40 mg/kg every 8 h and amikacin at a dosage of 15 mg/kg every 24 h). The reservoir was replaced after 5 days of therapy and it was obtained a negative result was obtained from the liquor culture after approximately 12 days, but a positive result in molecular tests persisted. The serial brain USs performed until 1 month and 15 days showed progressive ventricular dilatation requiring the positioning of a ventriculoperitoneal shunt (Figure 3C). At the follow-up US performed at 2 months of age, the patient presented further ventricular dilatation and intraventricular hyperechoic tissue (Figure 3D). Therefore, a brain MRI was performed at 2 months and 1 week of age, confirming the increased dilatation of the ventricles, which were filled with purulent material (Figure 3E,F). After 24 days, due to a malfunction of the second catheter from which periodic cerebrospinal fluid samples were taken, it was necessary to perform a second replacement. The persistent purulent CSF, worsening ventriculitis and ventricular dilatation, persistent positivity of molecular tests (film array), despite two catheter replacements and maximal systemic therapy (continuous infusion of meropenem), indicated refractory Gram-negative meningitis with ventriculitis. After 31 days of meropenem and 17 days of amikacin, given the poor CNS penetration of systemic agents under these conditions, intrathecal colistin was initiated and increased by 1 mg per day from 1 mg/day to 5 mg/day and later increased to 10 mg/day, administered for a total of 21 days. Combined systemic therapy with intravenous colistin (2.5 mg/kg every 12 h) and ceftazidime–avibactam (30 mg/kg every 8 h) was administered for 28 days. CSF sterilization was achieved after 14 days of combined intrathecal and systemic treatment. Despite microbiologic clearance, the infant’s neurological status remained profoundly impaired, with severe axial dystonia, dysphagia (which required the placement of a gastrostomy), absence of visual and auditory responses, ongoing seizures, and markedly abnormal EEG. Evoked potential testing showed the absence of cortical responses across auditory, visual, and somatosensory pathways, indicating diffuse and severe neurological injury. The follow-up MRI performed at 4 months showed a marked decrease in ventricular size, with septate ventricles containing residual purulent material, associated with diffuse white-matter hyperintensity and cystic degeneration. Despite aggressive medical and neurosurgical management (consisting of neuro-endoscopic multiple septectomies and placement of a new intraventricular shunt system), the infant developed severe neurological impairment, including multifocal slow and epileptiform abnormalities, axial dystonia, dysphagia requiring gastrostomy, and absent cortical responses on evoked potential testing. At the last neurological follow-up, the patient showed profound neurodevelopmental impairment.

**Figure 4:**
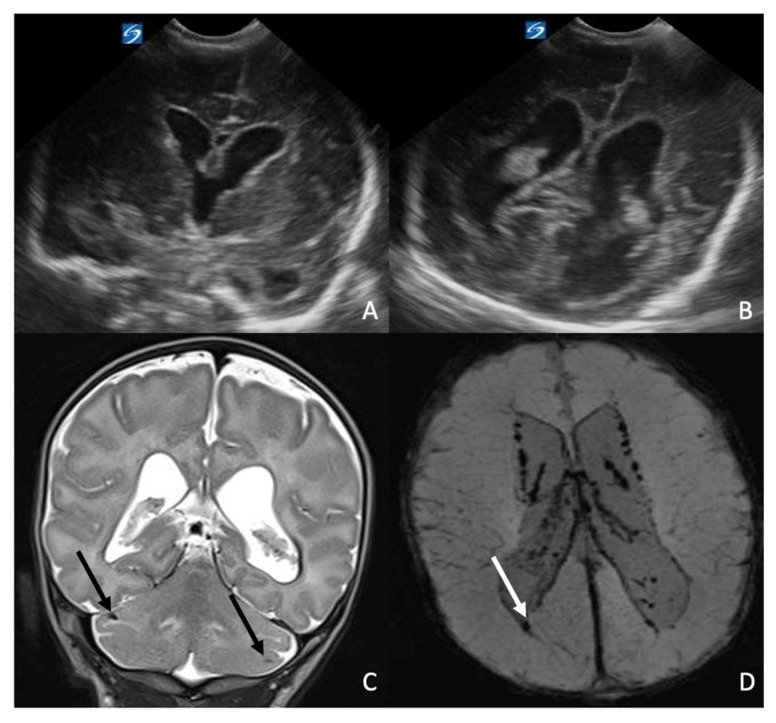
The US performed at 15 days of life (**A**,**B**) and an MRI performed at 20 days of life in a girl diagnosed with *Listeria monocytogenes* infection. The coronal US images (**A**,**B**) show mild asymmetry of the lateral ventricles with slight left predominance, associated with third-ventricle dilatation and diffusely hyperechoic ependyma, consistent with ventriculitis and prior subependymal haemorrhage, as well as increased echogenicity of the periventricular white matter. The subsequent brain MR, particularly coronal T2WI (**C**) and axial SWI (**D**), confirms subependymal haemorrhage, seen as irregular hypointensity along the ventricular walls, and shows associated mild intraventricular haemorrhage (white arrow in (**D**)) and cerebellar haemorrhagic foci (black arrows in (**C**)).

**Case 4:** The fourth patient was a 31-week premature female born via spontaneous vaginal delivery with Apgar scores of 4 and 6 at 1 and 5 min, respectively. Birth weight was 1970 g. The pregnancy was complicated by gestational diabetes requiring insulin therapy. Maternal infectious screening during pregnancy was negative. Due to meconium-stained fluid and cardiorespiratory depression at birth, the neonate required intubation and positive airway pressure ventilation for 24 h, followed by nasal CPAP for an additional three days. At birth, the infant presented with fever and elevated inflammatory markers. Broad-spectrum antibiotic therapy with ampicillin (100 mg/kg every 8 h) and netilmicin (3 mg/kg every 12 h) was started. Blood cultures, surface swabs, and bronchial aspirate were positive for *Listeria monocytogenes*; consequently, netilmicin was replaced with gentamicin (4 mg/kg every 24 h). A follow-up blood culture after four days of therapy was negative. A CSF analysis was initially deferred due to severe thrombocytopenia. Following the resolution of thrombocytopenia, a lumbar puncture performed 11 days later yielded negative culture and molecular results. However, a maternal swab performed at delivery subsequently tested positive for *Listeria monocytogenes*. At 24 h of life, a cranial ultrasound showed hyper echogenicity of the periventricular white matter. The patient underwent a brain US at 15 days of life, showing mild asymmetric dilatation of the lateral ventricles with left-sided predominance and mild dilatation of the third ventricle. The ependymal lining appeared diffusely hyperechoic, consistent with ventriculitis and prior subependymal hemorrhage, with associated increased echogenicity of the periventricular white matter (Figure 4A,B). At 16 days of age, the infant was transferred to our hospital for neurosurgical evaluation of hydrocephalus. The brain MRI performed at 20 days of life confirmed asymmetric supratentorial ventriculomegaly with left predominance, subependymal and intraventricular hemorrhage, and multiple hemorrhagic foci involving the germinal matrix, deep and periventricular white matter, and the cerebellum (Figure 4C,D). Given the triventricular dilation, serial brain US monitoring was instituted and a second MRI at 3 months of age was planned. Hydrocephalus findings remained stable, and the neurosurgical team determined that surgical intervention was not required. EEG results were normal. After initial feeding difficulties, the infant achieved adequate oral intake and satisfactory weight gain, without exhibiting neurological deficits. Ampicillin therapy was administered for a total of four weeks. Neurological and neuro-rehabilitative follow-up is currently ongoing. At the last evaluation, at 6 months of life, the neurological examination was normal for age.

## 3. Methods

This manuscript includes a case series and a narrative review of the literature. The case series was conducted and reported in accordance with the CARE (CAse REport) guidelines. The literature review was performed with the aim of contextualizing the presented cases and summarizing previously reported cases of congenital or neonatal *Listeria monocytogenes* infection with central nervous system involvement. We collected and summarized data on neonatal characteristics, neuroimaging, neurological involvement and procedures.

## 4. Results

Table 1 summarizes published cases of neonatal listeriosis. Across the published literature, we identified 23 reports describing congenital/neonatal *Listeria monocytogenes* infection with neurological and/or neuroradiological assessment, for a total of 37 patients [11,12,13,14,15,16,17,18,19,20,21,22,23,24,25,26,27,28,29,30,31,32,33]. When combined with our 4 additional neonates, the overall sample comprised 41 patients.

### 4.1. Perinatal Characteristics

Gestational age ranged widely from 29 to 40 weeks, with 27/41 (66%) infants born preterm (<37 weeks). Birthweight was variably reported, ranging from 775 to 3390 g, with missing data in several cases. Mode of delivery was inconsistently documented across reports; both vaginal delivery and caesarean section were represented, reflecting heterogeneous obstetric contexts (including emergency deliveries due to maternal infection or fetal distress).

Maternal history was frequently notable for fever and/or influenza-like or gastrointestinal symptoms in the days preceding delivery, and maternal antibiotic treatment was often administered when infection was suspected. Microbiological evidence of maternal infection was reported in some cases (e.g., positive vaginal swabs, maternal blood cultures, and/or placental cultures).

### 4.2. Clinical Presentation and Neonatal Management

Most infants presented with an early-onset sepsis phenotype, often accompanied by respiratory distress/failure and, in a subset, neurological signs such as hypotonia and seizures. Advanced cardiorespiratory support was occasionally required, including inotropes, inhaled nitric oxide, and ECMO in severe cases. Antimicrobial management most commonly consisted of ampicillin-based regimens, frequently combined with gentamicin, with treatment duration ranging from approximately 14 to 21 days when specified, and longer courses in complicated CNS disease.

**Table 1:** Literature report on neonatal listeriosis from 2000 ongoing compared to our cases. CNS: central nervous system; CSF: cerebrospinal fluid; CT: computed tomography; DOL: day of life; EEG: electroencephalography; GI: gastro-intestinal; MRI: magnetic resonance imaging; US: ultrasound; VP: ventriculoperitonea.

Author, Year	N.	Gestational Age (Weeks)/Birthweight (g)	Preterm Birth	Mode of Delivery	Maternal History and Management	Neonatal Clinical Features	Neonatal Management	Microbiological Data	Neuroimaging Neurological Outcome
Ramdani-Bouguessa et al., 2000 [11]	2	38–39/3300–2850	0	Vaginal	Green vaginal discharge 1 week before delivery/fever 2 days before delivery	Respiratory distress syndrome and neonatal sepsis/Convulsions	Ampicillin+ gentamicin 2/2 (100%)	*Listeria monocytogenes* (maternal vaginal secretions 2/2 neonatal blood, urine 2/2, CSF 1/2)	Normal: 1/2 (50%); Death: 1/2 (50%)
Benshushan et al., 2002 [12]	11	29–39/1300–3390	7/11 (64%)	Vaginal: 6 (55%); C-section: 4 (36%); Abortion: 2 (18%)	Maternal fever/sepsis; maternal antibiotics: 11/11 (100%)	Neonatal sepsis: 4/11 (36%); CNS involvement (meningitis and/or neurological signs: hypotonia, apnea) 3/11 (27%)	Neonatal antibiotics: 4/11 (36%)	*Listeria monocytogenes* (maternal blood/placenta; neonatal blood/CSF)	Normal: 7/11 (64%); Multiorgan failure and death: 1/11 (9%); NA (abortion): 2/11 (18%)
Chen et al., 2003 [13]	1	31/1070	1	Vaginal	Negative	Respiratory distress syndrome, asphyxia and suspected sepsis	Ampicillin and gentamicin for 18 days	*Listeria monocytogenes* (neonatal blood)	etNormal
Chen et al., 2007 [14]	2	31–28/1550–1180	2 (100%)	C-section /Vaginal	Maternal fever 2/2 (100%)	Erythematous maculopapular rash, neonatal sepsis/severe neonatal sepsis	Ampicillin + gentamicin 2/2 (100%)	*Listeria monocytogenes* (neonatal blood, and CSF ½ only blood 1/2)	Ventriculoperitoneal shunt on the 49th DOL + EEG with focal seizure 1/2 (50%) Death 1/2 (50%)
Vincent et al., 2009 [15]	1	33/2330	1	C-section	Fever	Hypotonia at birth	Ampicillin and gentamicin for 14 days, inotropic and anticonvulsant therapy	*Listeria monocytogenes* (neonatal blood, maternal blood and placenta)	Brain US: mild periventricular edema, discharged home at 30 DOL. No follow up
Mokta et al., 2010 [16]	1	40/3000	0	Vaginal	Negative	Fever, irritability, excessive cry, non-acceptance of feed and skin rash	Aeftriaxone (single dose)	*Listeria monocytogenes* (Neonatal blood and CSF, maternal vaginal and cervical swabs)	Death
Teixeira et al., 2011 [17]	1	25/775	1	Vaginal	Fever few days prior to delivery	Nonconfluent erythematous maculopapular and micro papular rash, septic shock with resuscitation at birth	Neonatal antibiotics surfactant, inotropes.	*Listeria monocytogenes* (placenta, neonatal blood was negative)	Death
Hong et al., 2012 [18]	1	34/2155	1	C-section	Maternal fever and gastro-intestinal symptoms	Respiratory distress and fever	Ampicillin for 14 days, gentamicin for 7 days	*Listeria monocytogenes* (amniotic fluid and neonatal blood)	Normal
Dinic et al., 2013 [19]	1	36/2350	1	Vaginal	Negative	Respiratory distress syndrome, hypotonia, thrombocytopenia, cutaneous rash and convulsions	Ampicillin	*Listeria monocytogenes* (neonatal blood, tracheal secretion and CSF)	Complete recovery, Normal
Charlier et al., 2014 [20]	2	31–36/NA	2	Vaginal 2/2 (100%)	Fever: 2 (100%), maternal antibiotics: 2 (100%)	Early-onset neonatal sepsis: 1 (50%); respiratory distress: 1 (50%)	Neonatal antibiotics (unspecified), 2/2 (100%)	*Listeria monocytogenes* (placenta 1/2, neonatal blood 1/2, gastric aspiration 2/2, CSF 1/2, ear swab 1/2)	Normal
Anand et al., 2016 [21]	1	40/NA	0	Vaginal	Negative	Fever, poor feeding, irritability and fussiness	Ampicillin and gentamicin for 21 days and acyclovir	*Listeria monocytogenes* (neonatal CSF)	MRI: mild enhancement of the leptomeninges At discharge: normal
Park et al., 2018 [22]	2	29–37/1800–3200	1/2 (50%)	C-section: 2/2 (100%)	Maternal fever/chorioamnionitis; antibiotics: 2/2 (100%)	Neonatal sepsis: 2/2 (100%); CNS involvement: 0/2 (0%)	Neonatal antibiotics: (unspecified) 2/2 (100%)	*Listeria monocytogenes* (maternal blood/placenta)	Normal: 1/2 (50%); Death: 1/2 (50%)
Luo et al., 2019 [23]	1	36/NA	1	C-section	Maternal fever	Neonatal sepsis	Ampicillin + gentamicin	*Listeria monocytogenes* (vaginal wabs, neonatal blood, CSF not obtained due to clinical instability)	Normal
Rabinowitz et al.,2020 [24]	1	36/2550	0	C-section	Maternal fever	Sepsis with respiratory failure and pulmonary hypertension	Ampicillin + gentamicin; iNO, inotropes and vasopressors, ECMO+ hemofiltration	*Listeria monocytogenes* (neonatal blood and placenta)	MRI: enlarged ventricle and mild edema of the with matter; Mild hypotonia and gross motor delay at 8 months of age
Rovas et al., 2022 [25]	1	36/2850	1	Vaginal	Fever and cough	Sepsis, congenital anemia and respiratory failure	Ampicillin + gentamicin; curosurf, inotropes	*Listeria monocytogenes* (neonatal blood and placenta)	Normal
Gomez et al., 2022 [26]	1	29/NA	1	C-section	Maternal fever and gastro-intestinal symptoms	Sepsis with respiratory failure and neurological involvement	Ampicillin 21 days + gentamicin 7 days	*Listeria monocytogenes* (neonatal blood, CSF not obtained due to clinical instability)	Hydrocephalus needing permanent ventriculoperitoneal shunt 3 years old: delayed speech and motor skills
Simão Raimundo et al., 2023 [27]	1	34/NA	1	C-section	Mild respiratory symptoms	Septic shock	Ampicillin + gentamicin	*Listeria monocytogenes* (neonatal blood)	Normal
Amano et al., 2023 [28]	1	35/2211	1	Vaginal	Maternal fever, reduced fetal movements	Neonatal sepsis	Ampicillin + gentamicin; IVIG; exchange transfusion for disseminated intravascular coagulation	*Listeria monocytogenes* (neonatal blood; maternal blood)	Normal
D’Aleo et al., 2024 [29]	1	31/NA	1	Vaginal	Maternal GI symptoms with reduced fetal movements	Neonatal infection without neurological signs	Ampicillin + gentamicin, 14 days	*Listeria monocytogenes* (cord blood, placental swabs)	Normal
D’sa et al, 2024 [30]	1	37/NA	1	Vaginal	History of fresh cheese assumption in the third trimester, no maternal symptoms	At 15 DOL fever and lethargy	Ampicillin 21 days + gentamicin, 7 days	*Listeria monocytogenes* (CSF fluid)	Brain MRI 1 month after discharge: hydrocephalus requiring VP shunt Last follow up at 15 months: normal neurological development + completely functioning VP shunt
Rodrigues Amaral et al., 2025 [31]	1	31/1545	1	C-section	Maternal fever	Neonatal sepsis and pneumonia with resuscitation at birth	Ampicillin + gentamicin, 14 days, cefotaxime 8 days	*Listeria monocytogenes* (neonatal blood; placenta)	MRI at term-equivalent age: Leukoencephalomalacia with ventricular dilatation At 7 months: mild axial and limb hypertonia
Tao et al., 2025 [32]	1	33/2395	1	Vaginal	Cough and fever	Asphyxia (hypotonia), severe respiratory distress with resuscitation at birth	Inotropic therapy, penicillin and cefoperazone/ sulbactam later replaced with penicillin and meropenem, acyclovir, blood transfusions and plasma infusions	*Listeria monocytogenes* (neonatal blood and CSF)	US: bilateral subependymal hemorrhage, CT: intraventricular and subarachnoid hemorrhage Death
Hu et al., 2025 [33]	1	33/NA	1	C-section	Fever postpartum; maternal antibiotics	Neonatal sepsis with respiratory distress	Piperacillin-tazobactam, then meropenem 18 days	*Listeria monocytogenes* (positive mother and neonate blood culture)	Normal
**Maddaloni et al., 2025**	4	39–37–37–31/3140–2830–3030–1870	1	C-section	Negative	1. sepsis with respiratory failure and generalized seizure 2. respiratory failure, pulmonary hypertension, cardiac disfunction 3.respiratory failure at birth and seizure. 4. cardiorespiratory failure at birth plus fever	1. ampicillin 21 days, gentamicin 14 days 2. ampicillin, gentamicin, ECMO, inotropes 3. ampicillin 21 days, gentamicin 14 days 4. ampicillin 4 weeks, gentamicin 14 days	*Listeria monocytogenes* 1. CSF culture 2. blood culture, CSF not performed for ECMO 3. blood and CSF culture 4. blood cultures, surface swabs and bronchial aspirate, CSF not performed for thrombocytopenia	1. Hydrocephalus requiring VP shunt. Normal neurological status 2. MRI 1 month: multiple cystic–hemorrhagic lesions within the bilateral periventricular white matter. Neurodevelopmental delay. 3. MRI: cerebral venous sinus thrombosis, intraventricular hemorrhage, tetraventricular hydrocephalus, ventriculitis and periventricular small abscess-like lesions. Need for neuroendoscopic multiple septostomies and placement of a new intraventricular shunt system Severe neurological damage. 4. MRI 20 DOL: dilation of the ventricular system associated with the presence of multiple hemorrhagic areas, some of which are cavitated. Normal neurological status.

### 4.3. Microbiological Confirmation

Microbiological confirmation was most commonly obtained from neonatal blood cultures, frequently supported by placental and/or maternal specimens (maternal blood, vaginal swabs). CSF culture confirmation was reported in a subset of cases; lumbar puncture was sometimes not performed or deferred due to clinical instability or severe thrombocytopenia. In our case series, *L. monocytogenes* was isolated from blood and/or CSF, and molecular testing contributed in selected cases.

### 4.4. Neuroradiological Findings

Neuroimaging findings were heterogeneous, ranging from mild periventricular edema or ventriculomegaly to severe patterns including hydrocephalus requiring ventriculoperitoneal shunting, cystic white matter injury/leuko-encephalomalacia, and intracranial hemorrhagic lesions. In our cohort, neuroimaging documented a broad spectrum, with progressive hydrocephalus in 3/4 infants and severe CNS involvement in one case complicated by refractory ventriculitis requiring intensive neurosurgical and antimicrobial management.

### 4.5. Neurodevelopmental Outcomes

Overall neurological outcome at discharge was variably reported: 22/41 (53.0%) were described as having a normal outcome, 7/41 (17.0%) had neurological impairment (including hydrocephalus requiring shunt and/or subsequent neurodevelopmental delay/seizures), and 7/41 (17%) died. Outcome data were not available in 5/41 (12%), largely due to pregnancy losses or lack of follow-up information.

## 5. Discussion

In this manuscript, we compared the clinical and neurological involvement observed in a series of newborns affected by congenital listeriosis with previously described cases in the literature. Our findings emphasize the importance of early identification of maternal risk factors and prompt diagnosis of *Listeria monocytogenes* infection during pregnancy, given the potential severity of neonatal outcomes and the availability of effective antimicrobial treatment.

### 5.1. Clinical Pictures, Diagnosis and Treatment

*Listeria monocytogenes* remains a rare but severe cause of early-onset (1–7 days) neonatal sepsis, with an estimated incidence of 0.2–0.7 per 1000 live births and a mortality rate up to 20%, showing symptoms similar to group B streptococcus infections [34]. Late-onset disease (days 8–28) more commonly presents with meningitis, may mimic viral encephalitis, and has lower mortality (20% vs. 60% compared to early-onset) [34].

Despite its relatively low frequency, the pathogen’s ability to cause fulminant fetal and neonatal disease makes maternal–neonatal listeriosis a major perinatal threat [35]. Vertical transmission typically results from maternal bacteremia, after which *Listeria* exhibits strong tropism for the placenta [36]. The placenta provides a privileged niche where the bacterium replicates efficiently and can invade fetal tissues, especially during the third trimester, when placental structure and immune dynamics favor bacterial translocation [37]. Through intracellular replication and actin-based motility, *Listeria* traverses trophoblastic layers and spreads hematogenous to the fetus, leading to systemic infection and CNS involvement [36].

This case series, aligned with previously described cases, illustrates the wide clinical spectrum of neonatal *Listeria monocytogenes* infection, ranging from full neurological recovery to severe, irreversible neurological injury. Our cases highlight how early-onset infection can rapidly progress to CNS involvement, hydrocephalus, and long-term neurodevelopmental impairment despite timely recognition and appropriate antimicrobial therapy. These observations are consistent with the previous literature indicating that neonatal listeriosis remains associated with substantial morbidity and mortality, especially when meningitis is present [10,14,21,32,38].

Early-onset neonatal listeriosis, as observed in our patient, typically manifests within the first 24–72 h of life, with respiratory distress, sepsis, neurological impairment, and seizures being common features of in utero infection. Cases 1 and 3 match this pattern, both evolving rapidly into severe CNS disease with hydrocephalus, ventriculitis, seizures, and the need for neurosurgical interventions. Neuroimaging abnormalities, including intraventricular hemorrhage, venous sinus thrombosis, abscess-like lesions, and multiloculated hydrocephalus, are well described in neonatal listeriosis and remain predictors of poorer neurological outcome [26,30,31,39,40]. In contrast, Case 2 demonstrated fulminant cardiorespiratory failure and refractory pulmonary hypertension requiring ECMO, a scenario already described in the literature [24,41].

Diagnosis relies on blood and CSF cultures, while molecular assays may accelerate confirmation [42]. However, lumbar puncture may be contraindicated in critically ill neonates, particularly those requiring ECMO or with coagulopathy. In such cases, neuroimaging and serial clinical evaluations play a key role in guiding therapy [43].

Recommended therapy remains ampicillin with an aminoglycoside, exploiting synergistic bactericidal activity and consistent susceptibility patterns, given for 10–14 days, or 14–21 days in cases of meningitis [43,44]. Severe CNS complications, however, may evolve despite appropriate therapy due to the organism’s neurotropism and the immature neonatal immune response, requiring prolonged and aggressive therapy [45].

### 5.2. Neuroradiological Findings

Neuroimaging is essential in neonatal meningoencephalitis to characterize the extent of infection and to guide therapeutic and neurosurgical decisions. While cranial ultrasound offers a rapid bedside assessment, MRI is the gold standard, providing superior delineation of ventriculitis, cerebritis, abscesses, venous thrombosis, and evolving hydrocephalus [46].

*Listeria monocytogenes* is responsible for purulent leptomeningitis [46]. The brain and meninges are affected by micro-abscesses and miliary granulomas [47]. A distinctive feature of *Listeria monocytogenes* meningoencephalitis is the chronic evolution of cystic encephalomalacia and periventricular cavitations, which are more frequently visible in the follow-up MRI of meningitis caused by this bacterium than others [47,48].

The imaging progression in our case—ventriculitis, intraventricular hemorrhage, venous sinus thrombosis, progressive and cystic encephalomalacia—mirrors the severe end of the spectrum as also described in the literature.

### 5.3. Neurodevelopmental Outcomes

Neonatal bacterial meningoencephalitis carries a high risk of long-term neurodevelopmental impairment [49]. Up to one third of survivors develop neurodevelopmental impairment, epilepsy, or motor deficits, particularly when early imaging shows white matter injury, hydrocephalus, or diffuse cystic changes [5,50].

Our cases reflect the heterogeneity of neurological outcomes: Case 1 shows near-normal function in adolescence despite early hydrocephalus, while Case 3 demonstrates severe, generalized neurologic disability. These contrasting outcomes emphasize the importance of long-term multidisciplinary follow-up, including neurology, neurosurgery, rehabilitation, audiology, and neurodevelopmental assessment.

### 5.4. Microbiological Resolution Versus Neurological Outcome in the Context of Intraventricular Devices

External ventricular drainage or access reservoirs are frequently needed for the management of post-infectious hydrocephalus, as also described in the literature [14,26,30]. However, CSF devices greatly raise the risk of nosocomial ventriculitis, especially because of multidrug-resistant (MDR) Gram-negative bacteria like ESBL-producing Klebsiella pneumoniae [51,52]. Prolonged hospitalization, repetitive catheter manipulation, and past antibiotic exposure all contribute to this risk, as seen by Case 3, which highlights the diagnostic and therapeutic issues given by nosocomial superinfection in the context of intraventricular devices.

Treatment of MDR Gram-negative ventriculitis is notoriously difficult because many systemic antibiotics exhibit poor CSF penetration, especially in the presence of dense purulent material or ventricular compartmentalization, even when administered via continuous infusion [44].

With reported CSF sterilization rates of 70–90%, including in neonates and infants, intrathecal or intraventricular antibiotic administration, particularly colistin, has emerged as a crucial salvage therapy for carbapenem-resistant Enterobacteriaceae CNS infections in refractory infections [53,54,55].

In Case 3, persistent ventriculitis despite prolonged antibiotic therapy (meropenem and amikacin) plus multiple catheter exchanges necessitated the initiation and gradual escalation of intrathecal colistin. Finally, the combination of intrathecal and systemic therapy ultimately achieved CSF sterilization, aligning with previous reports [43]. Nonetheless, the patient’s irreversible neurological deterioration demonstrates that microbiological treatment does not always translate into functional recovery when severe structural CNS damage occurs before efficient pathogen clearance.

### 5.5. Strengths and Limitations

From a clinical perspective, our findings reinforce the need for a high index of suspicion for congenital listeriosis in neonates presenting with early-onset sepsis and neurological signs, even in the absence of clear maternal risk factors or early imaging abnormalities. Early diagnosis, close neurological monitoring, and multidisciplinary management are essential to optimize outcomes and provide accurate counseling to families.

This study has several limitations. The small number of cases and the retrospective nature of both our series and the reviewed literature limit the generalizability of the findings. Reporting bias and incomplete clinical or follow-up data are inherent to case reports and case series, particularly with respect to long-term neurological outcomes. Outcome rates from our case series and the reviewed literature are not generalizable and should be interpreted with caution. We emphasized the need for larger, population-based studies to obtain more robust epidemiological estimates. Nonetheless, by integrating detailed clinical and neuroradiological data from our patients with previously published cases, this study contributes to a more comprehensive understanding of the neurological spectrum of congenital *Listeria monocytogenes* infection.

## 6. Conclusions

Our case series highlights that *Listeria monocytogenes* remains a severe neonatal pathogen, as maternal bacteremia and placental tropism may lead to significant CNS injury despite early antimicrobial therapy. Non-specific maternal and neonatal symptoms should prompt consideration of listeriosis, a thorough maternal history including dietary exposure, and timely microbiological investigations. Neuroimaging, particularly MRI, is crucial for detecting ventriculitis, hydrocephalus, and associated complications. The need for CSF devices increases the risk of nosocomial multidrug-resistant infections, which may require advanced intrathecal or intraventricular antibiotic therapy as salvage therapy. Consequently, severe early infection followed by MDR superinfection can result in profound neurodevelopmental impairment.

Despite advances in neonatal care, neonatal listeriosis continues to be associated with a high burden of long-term neurological sequelae, highlighting the need for improved preventive strategies, earlier maternal diagnosis, and optimized neonatal management.

## Data Availability

The original contributions presented in this study are included in the article. Further inquiries can be directed to the corresponding author.

## Abbreviations

The following abbreviations are used in this manuscript:

BAEP	Brainstem auditory evoked potential
CPAP	Continuous positive airway pressure
CNS	Central nervous system
CSF	Cerebrospinal fluid
CT	Computed tomography
DOL	Day of life
ECMO	Extracorporeal membrane oxygenation
EEG	Electroencephalogram
HFNC	High flow nasal cannule
MRI	Magnetic resonance imaging
NICU	Neonatal Intensive Care Unit
SSEPs	Somatosensory Evoked Potentials
